# Retinotopic and topographic analyses with gaze restriction for steady-state visual evoked potentials

**DOI:** 10.1038/s41598-019-41158-5

**Published:** 2019-03-14

**Authors:** Nannan Zhang, Yadong Liu, Erwei Yin, Baosong Deng, Lu Cao, Jun Jiang, Zongtan Zhou, Dewen Hu

**Affiliations:** 1 College of Mechatronic Engineering and Automation, National University of Defense Technology, Changsha, Hunan 410073 P. R. China; 2 Unmanned Systems Research Center, National Institute of Defense Technology Innovation, Academy of Military Sciences China, Beijing, 100081 P. R. China; 3 Air Force Engineering University, Xi’an, Shanxi 710051 P. R. China; 4 Tianjin Artificial Intelligence Innovation Center (TAIIC), Tianjin, 300450 China

## Abstract

Although the mechanisms of steady-state visual evoked potentials (SSVEPs) have been well studied, none of them have been implemented with strictly experimental conditions. Our objective was to create an ideal observer condition to exploit the features of SSVEPs. We present here an electroencephalographic (EEG) eye tracking experimental paradigm that provides biofeedback for gaze restriction during the visual stimulation. Specifically, we designed an EEG eye tracking synchronous data recording system for successful trial selection. Forty-six periodic flickers within a visual field of 11.5° were successively presented to evoke SSVEP responses, and online biofeedback based on an eye tracker was provided for gaze restriction. For eight participants, SSVEP responses in the visual field and topographic maps from full-brain EEG were plotted and analyzed. The experimental results indicated that the optimal visual flicking arrangement to boost SSVEPs should include the features of circular stimuli within a 4–6° spatial distance and increased stimulus area below the fixation point. These findings provide a basis for determining stimulus parameters for neural engineering studies, e.g. SSVEP-based brain-computer interface (BCI) designs. The proposed experimental paradigm could also provide a precise framework for future SSVEP-related studies.

## Introduction

Steady-state visual evoked potentials (SSVEPs) are periodic visual cortical responses evoked by certain repetitive stimuli with a constant frequency and can be detected from the primary visual cortex^1,2^. SSVEPs have many applications in neural engineering and neuroscience. Evidence suggests SSVEP is a robust method to study visual perception, spatial and selective attention, cognitive fatigue, working memory, and brain-computer interfaces (BCIs)^3–5^.

In particular, BCIs are communication technologies that can directly translate brain activity into commands to control external devices without the involvement of peripheral nerves and muscles^6,7^. Because of the high information transfer rate, minimal user training requirements, and simple system configuration, SSVEP BCIs have become one of the most promising paradigms for practical BCI applications^8,9^. An increasing number of researchers in the field of BCI have dedicated themselves to developing optimal SSVEP-BCI designs that will make SSVEP BCIs practical for communication and control. Thus far, researches have mainly focused on signal processing algorithms^10,11^, stimulus presentation paradigms^12,13^, and hybrid BCIs^14,15^. Only a few studies were committed to stimulus property selection^16^. Till now, retinotopic and topographic analyses of SSVEP have rarely been used for selecting optimal stimulus properties for SSVEP-BCI design.

Fuchs *et al*. investigated competitive neuronal dynamics of early visual processing and found that SSVEP amplitude elicited by stationary stimuli decreased significantly when competing stimulus flickers were in close spatial proximity within a visual angle of approximately 4.5° or less^17^. These experimental results provide a criterion for the minimal distance between adjacent stimulus flickers in SSVEP-BCIs. Vanegas *et al*. proposed a novel stimulus paradigm to improve the SSVEP signal-to-noise ratio (SNR) by exploiting individual primary visual cortex geometries^18^. In that study, SSVEPs were significantly enhanced by only manipulating the relative phase of the flicker among segments of a fovea-centered stimulus. Maye *et al*. introduced a new type of SSVEP-BCI paradigm that used only a single flicker stimulus and afforded multi-channel control^19^. The retinotopic map showed that by overtly directing attention to different flickers, the stimulus was projected to different positions on the retina. In almost all SSVEP-based BCI studies, the subjects needed to focus, fixate or track the visual stimuli according to the intended action. However, to our knowledge, no SSVEP studies have systemically performed retinotopic or topographic analyses with gaze restriction, which is hypothesized to be an important factor affecting the performance of SSVEP-BCIs.

The aim of this paper was to investigate the mechanisms of SSVEPs by incorporating retinotopic and topographic analyses under strictly experimental conditions. In our approach, we first designed an EEG eye tracking synchronous data recording paradigm for SSVEP studies. Specifically, forty-six flickers covering a visual field over an angle of 11.5° were successively delivered to evoke SSVEP responses in a random sequence. Biofeedback based on the eye tracker was provided for gaze restriction during visual stimulation. Second, the SSVEP responses in the visual field and topographic maps from full-brain EEG were plotted and analyzed. Third, recommendations for optimal SSVEP flicking arrangement to boost BCIs were discussed based on the experimental results.

## Results

### Distribution of SSVEP responses in the visual field

**Figure 1:**
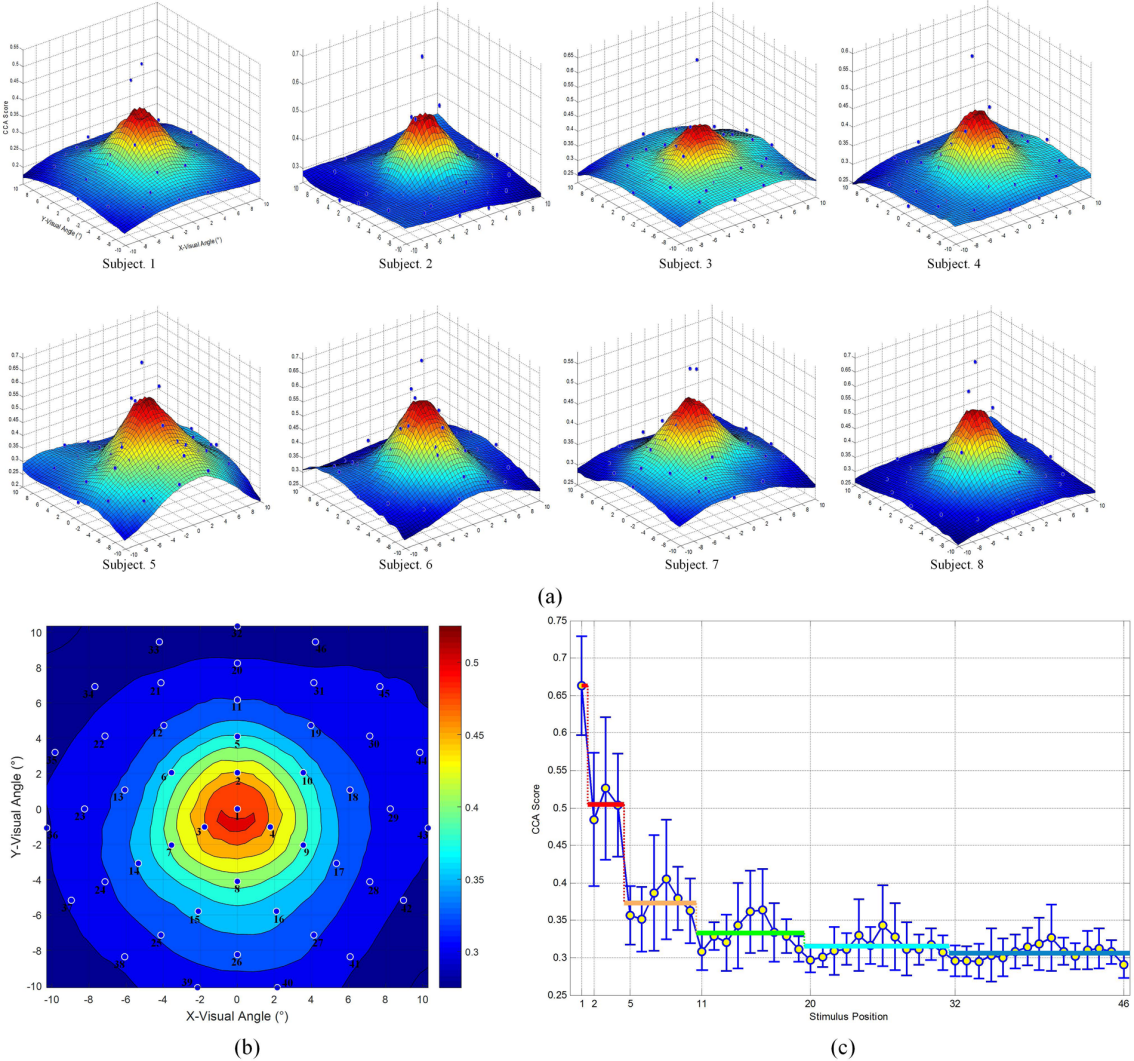
Distribution of SSVEP responses in the visual field. Panel (a) shows the three-dimensional shaded surface for each participant; (**b**) is referenced to the color bar, which depicts the average SSVEP response of all participants; the labels of all stimuli are provided in Fig. 5; (**c**) shows the average SSVEP response of each layer for all participants.

Figure 1 depicts the distribution of SSVEP responses in the visual field. Specifically, we first calculated the SSVEP responses of each position in the visual field for each participant and then successively plotted a three-dimensional shaded surface for each participant using a linear regression approach (see Fig. 1(a)). Here, the SSVEP responses were computed by the CCA algorithm using eleven channels over the occipital and parietal brain areas (P7, P3, Pz, P4, P8, PO3, POz, PO4, O1, Oz, and O2). Although the distributions of SSVEP responses varied across participants, they generally formed a Gaussian envelope, which corroborates the findings of previous visual cortex studies^20,21^. Furthermore, as shown in Fig. 1(b), we averaged the SSVEP responses over all participants. The average SSVEP response presented circular areas and exhibited a downward trend with increasing distance from the central visual field. Interestingly, the SSVEP responses were generally higher below the horizontal midline of the visual field than above the horizontal midline of the visual field. In addition, the average SSVEP responses of each layer for all participants are shown in Fig. 1(c). The results suggest that the average SSVEP response declined dramatically within the first three layers (0–4°) but declined slowly within the last three layers (6–10°).

In SSVEP BCIs, the target recognition accuracy is usually affected by the peripheral stimuli around the central stimulus. To further evaluate the competitive effect between central and peripheral stimuli, we calculated the percentage of SSVEP responses to the peripheral stimuli (stimuli in layers 2–6) that were higher than SSVEP responses to the central stimulus, which was defined as the error rate. The two-sample *t*-test was performed for further statistical comparisons of the SSVEP responses. The error rates and *p*-values are summarized in Table 1. Specifically, the average error rate was 11.55% (*p* > 0.001) at 2° (layer 2), decreased to 5.30% (*p* < 0.001) at 4° (layer 3), and ultimately reached 2.08% (*p* < 0.001) at 6°. We note that these findings are in line with the results of Fig. 1(c).

**Table 1:** Competitive effect between central stimuli and peripheral stimuli.

Participant	Visual Angle				
	2°	4°	6°	8°	10°
S1	[25.00%, 0.0057]	[8.33%, <0.001]	[8.33%, <0.001]	[8.33%, <0.001]	[8.33%, <0.001]
S2	[8.33%, <0.001]	[0, <0.001]	[0, <0.001]	[0, <0.001]	[0, <0.001]
S3	[0, <0.001]	[0, <0.001]	[0, <0.001]	[0, <0.001]	[0, <0.001]
S4	[8.33%, <0.001]	[8.33%, <0.001]	[0, <0.001]	[0, <0.001]	[0, <0.001]
S5	[9.09%, 0.0061]	[9.09%, <0.001]	[0, <0.001]	[0, <0.001]	[0, <0.001]
S6	[8.33%, 0.0019]	[8.33%, <0.001]	[0, <0.001]	[0, <0.001]	[0, <0.001]
S7	[25.00%, 0.0182]	[0, <0.001]	[0, <0.001]	[0, <0.001]	[0, <0.001]
S8	[8.33%, 0.0033]	[8.33%, <0.001]	[8.33%, <0.001]	[8.33%, <0.001]	[8.33%, <0.001]
AVE	[11.55%, <0.001]	[5.30%, <0.001]	[2.08%, <0.001]	[2.08%, <0.001]	[2.08%, <0.001]

The competitive effect is depicted by [error rate, *p*-value], in which the error rate is defined as the percentage of SSVEP responses to the peripheral stimuli (stimuli in layers 2–6) that were higher than SSVEP responses to the central stimulus, and the *p*-value was calculated by the two-sample *t*-test.

### Effects of stimulus position on SSVEP responses

**Figure 2:**
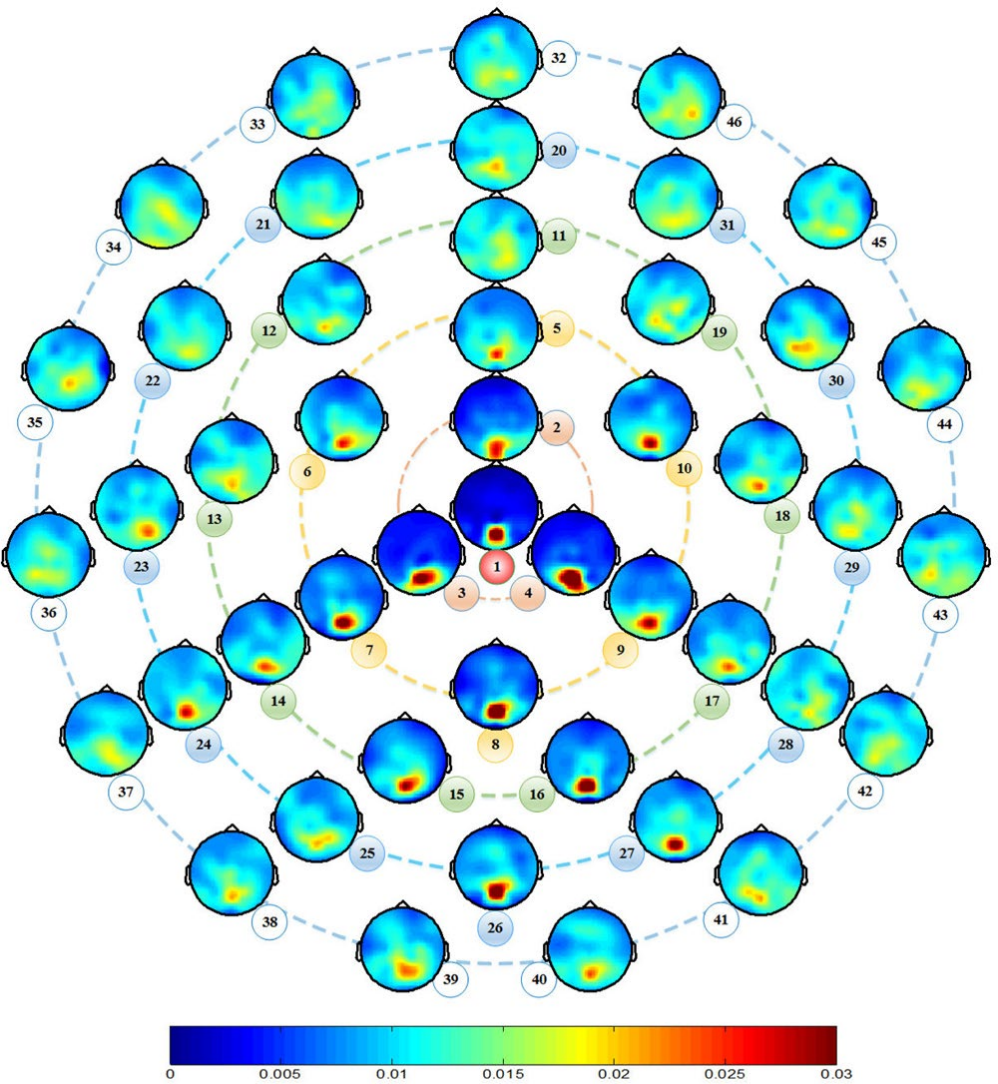
Topographic maps of channel contributions to SSVEP detection. The numbers denote the indexes of the visual stimuli. Refer to Figs 5 and 6 for the locations of the visual stimuli and the names of the full-brain electrode locations, respectively.

The effects of stimulus position on SSVEP responses were also evident in the topographical maps based on the full-brain EEG analyses. As shown in Fig. 2, the occipital region had the highest contribution to SSVEP detection compared with other brain regions, which further supports the conclusion that SSVEP are produced mainly from the primary visual cortex^22^. Here, the channel contributions were calculated using formula 4. Importantly, the stimuli that were closer to the visual field center evoked stronger SSVEP responses, and the stimuli below the horizontal midline of the visual field evoked stronger SSVEP responses than above the horizontal midline of the visual field, which is in line with the results shown in Fig. 1. In addition, we found the largest SSVEPs were recorded from the occipital scalp sites, contralateral to the stimulus position in the horizontal direction, and these SSVEPs remained generally positive for all stimulus positions. For instance, when the visual stimulus was delivered to position 4 (right side of the visual field), the corresponding SSVEP responses recorded from channels on the left side were stronger than those recorded from the right side. This phenomenon is known as the contralateral effect^23,24^.

To further quantify the contralateral effect, we selected four channel pairs (i.e., O1–O2, PO3–PO4, P3–P4, P7–P8) in the occipital region to compare the SSVEP scores between hemifields (see formula 5). We first calculated the average scores of the total stimuli within certain degrees (i.e. 2°, 4°, 6°, 8°, or 10°) for the left and right channels. After that, the Kruskal-Wallis (KW) test, instead of the *t*-test, was performed for further statistical comparisons because the samples were not normally distributed. The scores of the channel pairs (see formula 5) together with the KW test results are summarized in Table 2. We found that the contralateral effect associated with SSVEPs was largest for the PO3–PO4 and P3–P4 channel pairs and showed significance within all visual angles. Similar findings were also reported by Clark *et al*.^24^. However, the contralateral effects at the O1–O2 and P7–P8 channel pairs were relatively lower and did not show significance in most cases. This finding can be intuitively understood for the case of the P7–P8 channel pair because of the low SSVEP response at these channels. In contrast, as shown in Fig. 2, the O1–O2 channel pair had the most significant SSVEP response evoked by the visual stimuli on both sides. These bilateral SSVEP responses may have occurred because these channels were located in the extrastriate (association) area, which also receives information from the ipsilateral visual field indirectly via the corpus callosum^23^.

**Table 2:** Scores of channel pairs and KW test results from the stimuli within certain visual angles over all participants.

Visual Angle	Channel Pair							
	O1–O2		PO3–PO4		P3–P4		P7–P8	
	Left	Right	Left	Right	Left	Right	Left	Right
2°	[+0.14, 0]	[−0.10, 0]	[−1.27, 1]	[+2.05, 1]	[−1.03, 1]	[+2.97, 1]	[−0.80, 1]	[+0.59, 1]
4°	[−0.03, 0]	[+0.06, 0]	[−0.86, 1]	[+1.16, 1]	[−0.54, 1]	[+1.95, 1]	[−0.50, 1]	[+0.91, 1]
6°	[−0.12, 1]	[+0.12, 0]	[−0.53, 1]	[+0.85, 1]	[+0.21, 1]	[+2.06, 1]	[−0.03, 0]	[+0.32, 0]
8°	[−0.10, 1]	[+0.11, 0]	[−0.49, 1]	[+0.63, 1]	[+0.38, 1]	[+1.92, 1]	[−0.13, 0]	[+0.21, 0]
10°	[−0.06, 1]	[+0.09, 0]	[−0.36, 1]	[+0.42, 1]	[+0.56, 1]	[+1.85, 1]	[−0.02, 0]	[+0.22, 0]

The significance level of the KW test was set to 0.05.

## Discussion

In this paper, we first proposed a novel experimental paradigm to exploit the features of SSVEPs. Specifically, an EEG eye tracking synchronous data recording system and biofeedback for gaze restriction were designed to create an ideal observer condition. Forty-six flickers covering a visual field over an angle of 11.5° were successively delivered to evoke SSVEP responses in a random sequence. Furthermore, the distribution of SSVEP responses in the visual field and the effects of stimulus position on SSVEP responses were comprehensive analyzed retinotopic and topographic maps. The optimal visual flicking arrangement to boost SSVEP-BCIs was further provided and discussed as below:

### Stimulus Shape

In our experimental results, we found that the average SSVEP responses in the visual field presented a circular pattern and showed a downward trend with increasing distance from the center (see Fig. 1(a and b)). This finding suggests that a circular stimulus may be more efficient for evoking SSVEPs than an equal-area stimulus with other shapes. Duszyk *et al*. performed a systematic comparison of SSVEP amplitude between circular stimuli and equal-area square stimuli across different stimulus frequencies^25^. The experimental results revealed that circular stimuli outperformed square stimuli in four out of the five stimulus frequencies, which further confirmed our speculation. There is always a tradeoff between the stimulus size and the number of visual stimuli on a certain screen. Hence, circular stimulus are likely optimal in SSVEP-BCI designs.

### Spatial Distance

A central stimulus can elicit more significant SSVEPs than a peripheral stimulus^26^. However, multiple stimuli delivered simultaneously to the visual field will influence the performance of SSVEP-BCI systems due to the limited visual neural resources. As shown in Fig. 1(a), some participants may have a wider visual field or could not evoke significant SSVEP responses. In that case, there may be no obvious difference between the SSVEP responses of the peripheral stimuli and that of central stimulus, as well as the EEG background activities can also lead the identification errors. Therefore, the spatial distance between the central stimulus (attended stimulus) and the peripheral stimulus (competing stimulus) becomes an important factor in SSVEP-BCI designs. In our study, the experimental results showed that the average SSVEP responses decreased dramatically within 4° but decreased less rapidly between 6°-and 10° (see Fig. 1(c)). Moreover, as shown in Table 1, the error rates caused by the peripheral stimuli decreased from 11.55% to 5.30% within 2–4° and were rather stable within 6–10° (maintained at 2.08%). Thus, we conclude that the center-to-center spatial distance between visual stimuli of SSVEP-BCIs should be more than 4° to avoid significant stimulus competition, and superior SSVEP-BCI accuracy could be attained when stimuli are placed spatially more than 6°. To achieve the tradeoff between the number of items filled in certain area and detection accuracy, the optimal center-to-center spatial distance between visual stimuli should be within 4–6°.

### Stimulus Position

**Figure 3:**
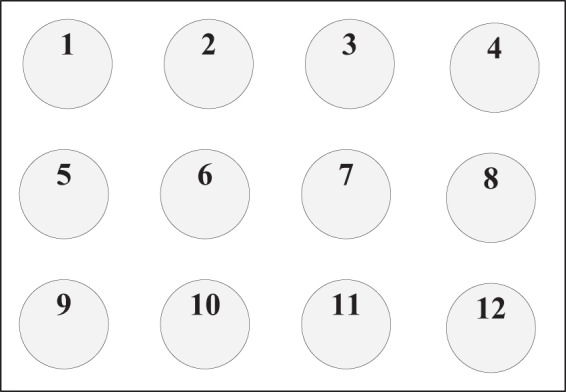
Suggested relative position between the characters and flickers.

In conventional SSVEP BCIs, the target characters are generally positioned in the center of the flickers. However, we found that stimuli below the horizontal midline of the visual field evoked much stronger SSVEP responses than those above the horizontal midline of the visual field, as shown in both Figs 1(b) and 2. These findings suggest that, to optimize the design of SSVEP-BCI systems, the characters on the visual stimuli should be positioned slightly higher to increase the stimulus area below the character (see Fig. 3). Therefore, when the participants automatically gaze on the characters, a stronger SSVEP response could be evoked.

### Customized Design

As shown in Fig. 1 and Table 1, the distribution of SSVEP responses varies amongst individuals. The proposed EEG eye tracking platform can be used to design an optometry instrument for selecting the optimal stimulus parameters for different users. The customized stimulus properties could help with the adaptive transformation of SSVEP-BCIs for each individuals.

### Future Work

To boost SSVEPs, this study analyzed the retinotopic and topographic of the visual stimuli from multiple discrete locations. The simple visual stimuli with same frequency and same phase were employed to evoke SSVEPs during the experiments. Vanegas *et al*. introduced a series of novel flicker-phase manipulations by integrating across subregions of a single stimulus, which resulted in a significant enhancement of SSVEP response^18^. This work is totally complementary to our current study, which leads more possible for the further researches in both neuroscience and SSVEP-BCIs perspectives. In our future work, based on the proposed EEG-eye-tracking experimental paradigm, we will focus on exploiting SSVEP response by considering both the flicker-phase manipulation of single stimulus and the multiple visual stimulus arrangement in terms of stimulus shape, distance and position.

## Methods

### Paradigm design

#### System structure

**Figure 4:**
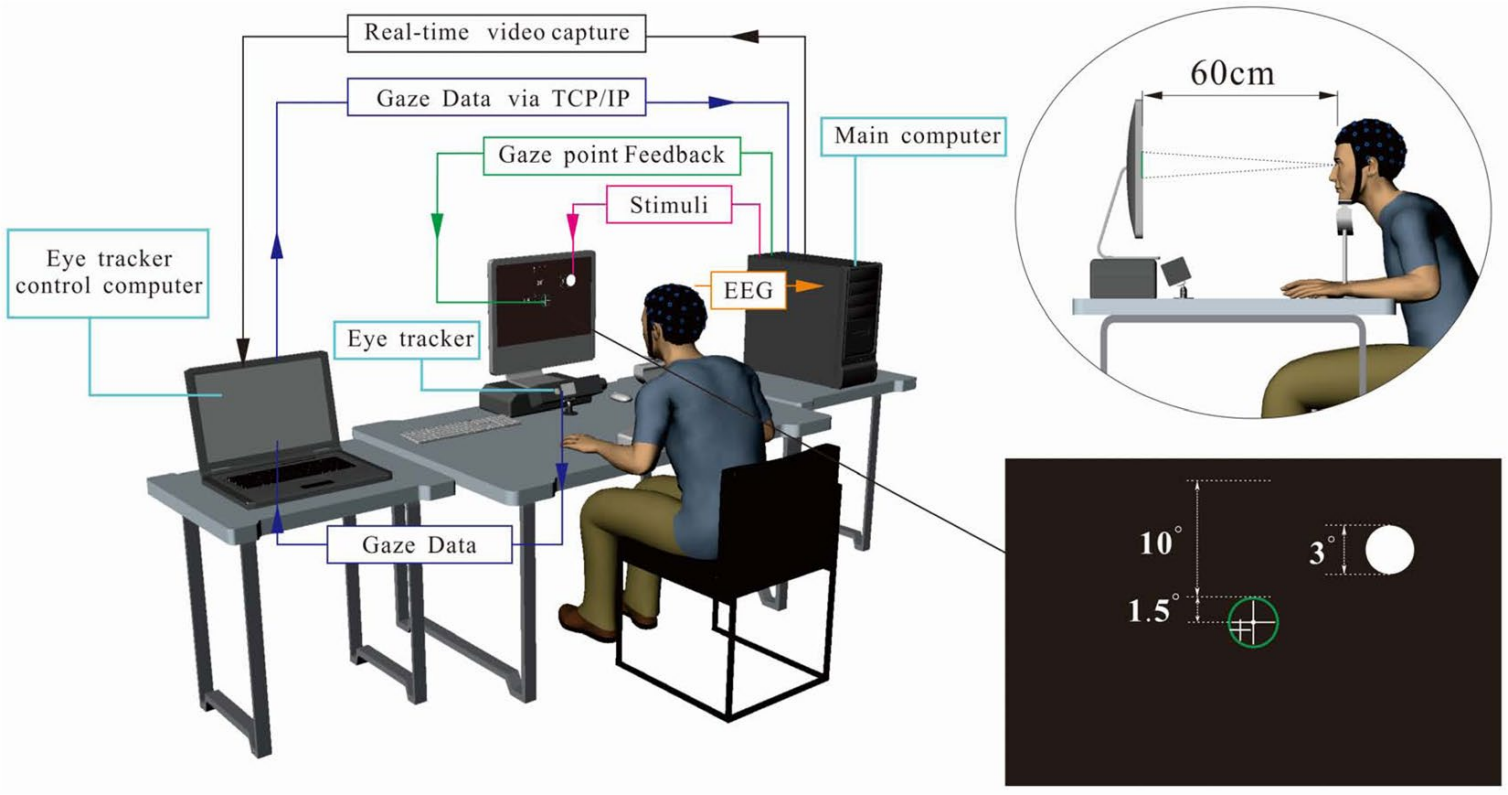
Schematic of the experimental system.

The experimental paradigm was designed using BCPy2000, a contribution of the BCI2000 platform^27^. In our approach, we used the Python interface of BCPy2000 for experimental procedure control, visual stimulus presentation and data recording (including gaze data and EEG data) in real-time. As shown in Fig. 4, the experimental system consisted of an eye tracker, an EEG data collection system, and a stimulus presentation system, which was implemented using two computers (i.e., a main computer and an eye tracker control computer). Here, the main computer was employed to control the visual stimulation, record EEG data, receive eye movement data from the eye tracker control computer for online gaze point feedback, and send the experimental monitoring video to the eye tracker control computer. The eye tracker control computer was used for collecting gaze data and sending data to the main computer in real time, while displaying the experimental monitoring video to the experimenter. Specifically, the eye movement data transmission between the computers was implemented via the TCP/IP protocol.

#### Visual stimulus mechanism

**Figure 5:**
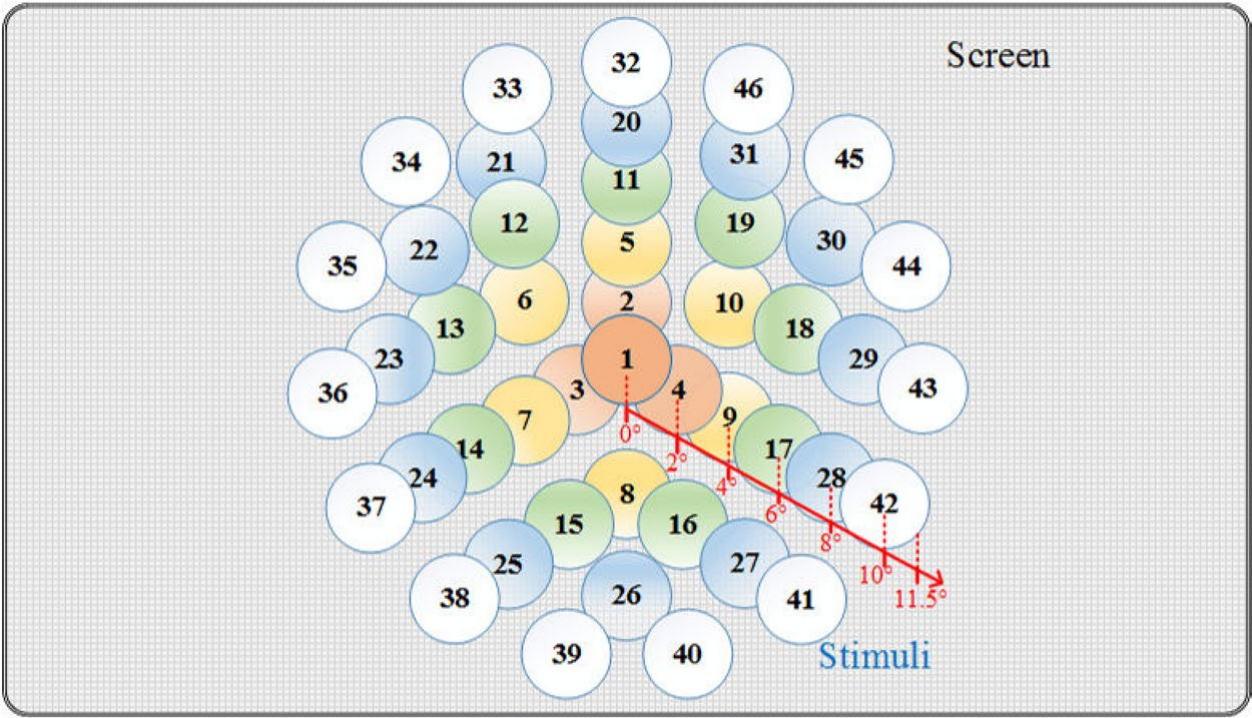
The distribution of the visual stimuli. The numbers and colors in the stimulus circles are only shown in this schematic for illustration and were not shown during the experiments.

In our approach, the visual stimulus was provided using a periodic flicker (a white circle) whose appearance and disappearance alternated on a black background with a duty of 50% (see Fig. 4). The frequency of the flicker was set at 15 Hz, corresponding to one-fourth of the refresh rate of the display monitor. The phase of the flicker was set to 0°. The flicker frequencies were generated by an independent timer programmed using Python, and synchronized with the main process through multithreading. The frequency selection was discussed thoroughly in^28^ and in our previous studies^29–31^. Moreover, the diameter of the flicker was set to a visual angle of 3° (3.14 cm). As shown in Fig. 5, forty-six flickering circles were successively delivered to evoke SSVEP responses in a random sequence. The distance between the center of the two adjacent layers corresponded to a visual angle of 2°, and all the flickers covered a visual field with an angle of 11.5° (six layers in total).

To investigate the effect of the visual stimulus with strict gaze fixation, the gaze points of the participants were displayed as a white cross in real time, and a green ring with a radius of 1.5° around the center of the monitor was employed to restrict the gaze points. Experimental trials were removed if any of the gaze points were beyond the green ring. During the experiment, the participants were required to rest their chin on a tabletop chinrest, which was adjusted to ensure that their eyes directly faced the center of the monitor.

### Experimental setup

#### Participants

The study was approved by local ethics committee of National University of Defense Technology. All experiments were performed in accordance with relevant guidelines and regulations. The experiments were conducted with eight healthy subjects (two females and six males, aged 23–30 years, with a mean age of 24.9 years). All participants had normal vision, and none had any uncorrected visual impairments or known cognitive deficits. None had previous experience with an eye tracker or the proposed visual stimulus paradigm. The participants were not allowed to drink any caffeine or alcohol in the four hours before each session. All participants provided written informed consent in accordance with the Declaration of Helsinki after the purpose of the study and the task required were explained in detail.

#### Data collection

**Figure 6:**
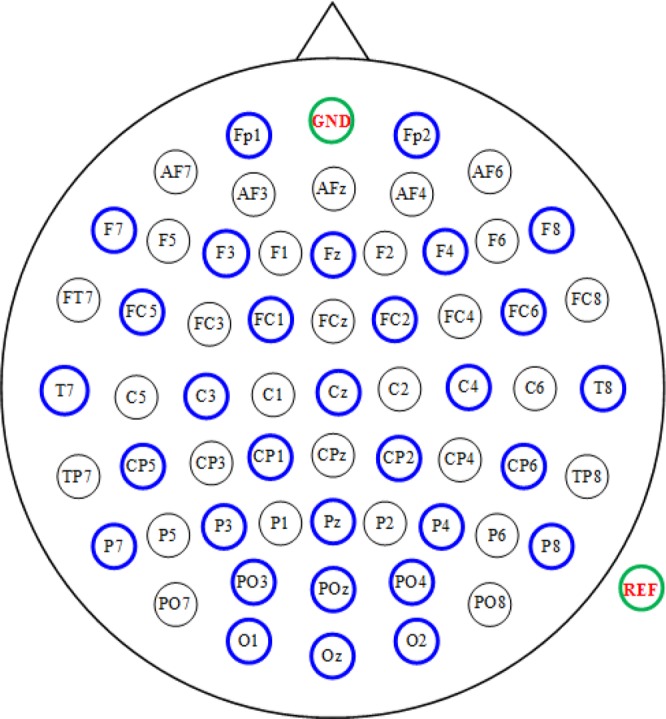
Configuration of the electrode locations used in this study.

EEG data were collected at a sampling rate of 500 Hz via a BrainAmp DC amplifier (Brain Products GmbH, Germany). As shown in Fig. 6, a total of 31 active electrodes were placed according to the extended international 10–20 system standard, grounded to FCz and referenced to TP10. Electrode impedances were kept below 10 kΩ.

Gaze data were recorded at a sampling rate of 60 Hz via an Eye-Trac6 eye tracker (ASL, USA). The eye tracker could track gaze over a vertical visual angle of approximately 30–35° and a horizontal visual angle of approximately 40–45°. Moreover, a nine-point calibration procedure was performed before data collection.

#### Experimental procedure

The experiments were performed in a quiet data collection room. The participants were seated 60 cm in front of a 19 inch LED monitor with a refresh rate of 60 Hz. The chair in which the participants were seated was adjusted for different individuals to ensure their chins were aligned with the chinrest. A short familiarization session was performed prior to initiation of the experiments.

**Figure 7:**
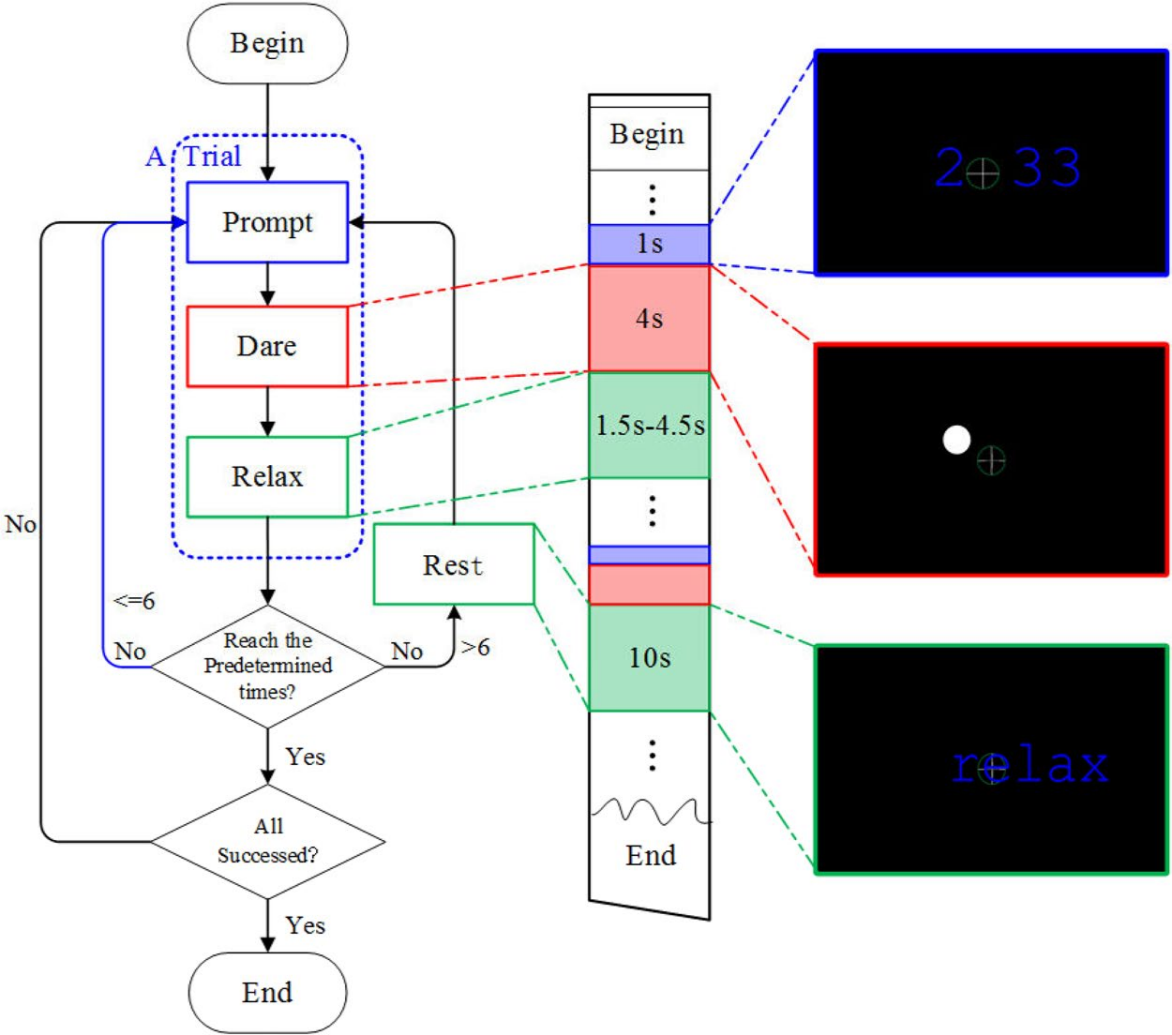
Illustration of the experimental process.

Each participant was requested to complete 12 runs on two half days within two days. In each run, the participants were asked to successfully complete 46 trials. As shown in Fig. 7, each trial consisted of three stages: Prompt, Dare, and Relax. In the Prompt stage, two Arabic numerals were displayed for 1 s: the number of trials until Rest and the number of remaining trials. These two numbers were displayed so that the participants could be aware of the progress of the current run in case they became bored with the procedure. Next, in the Dare stage, one of the circles was flashed at the specified frequency for 4 s to evoke SSVEPs. During the Dare stage, instead of gazing at the flicker, the participants were instructed to maintain their gaze on the center of the monitor. Real-time feedback of their gaze point was provided to allow the participants to control their gaze. The challenge was to maintain their gaze in the green ring during the Dare stage. In our approach, a successful trial was defined as none of the gaze points being located beyond the green ring during the Dare stage. In the Relax stage, the participants were allowed to rest for 1.5–4.5 s to mitigate fatigue. During the experiments, every six trials were followed by a 10-s Rest stage to allow the participants to take a break. The principal objective of this study was to create an ideal observer condition to exploit the features of SSVEPs rather than to build a full BCI system. To ensure the EEG data were collected under strict gaze fixation, the participants were asked to keep their eyes focused on a fixation cross on the center of screen during the “Dare stage” instead of directly focused on the visual stimuli like common BCI experiments, and a successful trial was defined as none of the gaze points being located beyond the green ring around the cross. The system automatically removed the failed trials and repeated them until all trials were successfully completed (see bottom left of Fig. 7). Additional details of the experimental design are illustrated in the Supplementary Video (see the Supplementary Data file available at http://ieeexplore.ieee.org).

### Data analyses

#### Pre-processing

The EEG data were treated with a 50-Hz notch filter and subsequently with a 4–35-Hz bandpass filter. The 4-s segment beginning at the stimulus onset was then extracted for SSVEP feature analysis.

#### Canonical correlation analysis

Canonical correlation analysis (CCA) is a statistical algorithm to measure the underlying correlation between two multidimensional variables^32^. Recently, CCA has been widely employed for frequency detection in SSVEP BCIs^33,34^. CCA is used to find the weight vectors *W*_*x*_ and *W*_*y*_ by maximizing the correlation between *x* and *y*, as follows:

1 $$\begin{array}{rcl}R & = & \mathop{{\rm{\max }}}\limits_{{W}_{x},{W}_{y}}\,\rho (x,y)\\  & = & \frac{E[{W}_{x}^{T}X{Y}^{T}{W}_{y}]}{\sqrt{E[{W}_{x}^{T}\cdot X{X}^{T}\cdot {W}_{x}]\cdot E[{W}_{y}^{T}\cdot Y{Y}^{T}\cdot {W}_{y}]}}\end{array}$$

2 $$x={X}^{T}{W}_{x},\,y={Y}^{T}{W}_{y}$$

where *R* is the CCA coefficient, *X* is the multi-channel EEG signal, and *Y* is the reference signal represented as

3 $${Y}_{f}=[\begin{array}{c}\sin \,(2\pi \cdot f\cdot t)\\ \cos \,(2\pi \cdot f\cdot t)\\ \sin \,(2\pi \cdot 2f\cdot t)\\ \cos \,(2\pi \cdot 2f\cdot t)\\ \vdots \\ \sin \,(2\pi \cdot Mf\cdot t)\\ \cos \,(2\pi \cdot Mf\cdot t)\end{array}],\,t=\frac{1}{S},\frac{2}{S},\ldots ,\frac{N}{S}$$

where *f* is the frequency, *M* is the number of harmonics (which *M* = 3 in this study), *N* is the number of sampling points per trial, and *S* is the sampling rate.

#### Single-channel contributions based on CCA coefficient

To illustrate the single-channel contributions to SSVEP detection, instead of using the CCA method directly, we calculated the single-channel contribution as the difference in the outcome of the CCA analysis with and without including the corresponding channel. A similar idea was introduced by Maye *et al*.^19^. The single channel contribution is defined as

4 $${r}_{i}=\frac{{\sum }_{j=1,2,\ldots i-1,i,i+1,\ldots C}^{C}\,{R}_{j}-{\sum }_{j=1,2,\ldots i-1,i+1,\ldots C}^{C}\,{R}_{j}}{{\sum }_{j=1,2,\ldots i-1,i,i+1,\ldots C}^{C}\,{R}_{j}}$$

where *i* is the index of the considered channel, *j* is the channel index, and *C* is the total number of EEG channels, i.e., 31 in this study.

#### Contralateral effect analysis

To investigate the contralateral effect of different visual stimuli in the visual field, we paired the symmetrical EEG channels (e.g., O1–O2, PO3–PO4, P3–P4, P7–P8; see Fig. 6) and compared the corresponding SSVEP responses. Subsequently, we also employed the SSVEP responses evoked by the central stimulus (i.e., stimulus 1; see Fig. 5) as a reference to reduce the variation between the left and right hemispheres. Finally, the score of the contralateral effect of the visual stimuli can be represented as

5 $${E}_{s,d,p}=\frac{avg\,({\sum }_{k\in K}\,{r}_{s,k,p\_left})}{{r}_{0,0,p\_left}}-\frac{avg\,({\sum }_{k\in K}\,{r}_{s,k,p\_right})}{{r}_{0,0,p\_right}}$$

where *s* is the side of the stimuli in the visual field (left or right side); *d* is the visual angle of the visual stimuli within *d* = 2°, 4°, 6°, 8°, or 10°, corresponding to the five layers of stimuli (see Fig. 5); *p* is the index of the channel pairs in the visual cortex (each pair includes *p*_*left* and *p*_*right* channels, e.g., O1–O2); *k* is the stimulus index, and *K* is the total number of stimuli within degree *d* in side *s*.

## Supplementary information

supplementary video

LaTeX Supplementary File
